# Skin Picking Disorder: Does A Person’s Sex Matter?

**DOI:** 10.12788/acp.0049

**Published:** 2022-02-01

**Authors:** Jon E. Grant, Samuel R. Chamberlain

**Affiliations:** 1 University of Chicago, Department of Psychiatry and Behavioral Neuroscience, Chicago, IL USA; 2 Department of Psychiatry, Faculty of Medicine, University of Southampton; and Southern Health NHS Foundation Trust, Southampton, UK

**Keywords:** gender, sex, skin picking disorder, excoriation, comorbidity, impulsivity

## Abstract

**Background:**

Skin picking disorder (SPD) is characterized by recurrent picking with scarring or tissue damage. Although research suggests that less than half of people with SPD are male, there is little clinical information about men with SPD.

**Methods:**

We recruited 95 non-treatment seeking adults as part of a cross-sectional study of SPD. Men (n=17) and women (n=78) with SPD were compared on clinical and cognitive measures. Sex differences in the demographic and clinical characteristics, skin picking sites, and presence of comorbidities, were examined using analysis of variance for continuous variables and likelihood ratio chi-square tests for categorical variables.

**Results:**

Men were significantly more likely than women to report a first-degree relative with skin picking or hair pulling. Men were less likely to pick from their scalps, backs, and breasts and picked from fewer sites. Men and women did not significantly differ on skin picking severity, disability, impulsivity, or quality of life.

**Conclusions:**

These data indicate that SPD is similarly impairing for men and women, but men may have higher familial loading and a somewhat different distribution and frequency of picking sites. Sex differences in SPD merit more detailed consideration in larger samples, including addressing potentially higher genetic/familial loading in males.

## Introduction

Skin-picking disorder (SPD) is characterized by recurrent skin picking resulting in tissue damage. In addition to the repetitive picking and associated skin damage, this disorder often leads to clinically significant impairment or distress (1). SPD is also often accompanied by increased anxiety, depression, and other psychosocial dysfunction (1–2).

A recent large community prevalence study (n=10,169 adults) representative of the general US population found that 2.1% identified as having current SPD, with just slightly less than half (44.6%) being male (3) (but see a recent, large Brazilian epidemiologic survey of 7639 people which found that 3.4% endorsed SPD of whom 82.2% were female) (4). Data regarding clinical presentations of SPD, neuroimaging, and treatment studies, however, have largely been conducted in females with SPD (e.g. 5-14), and so these new prevalence data (i.e. that approximately half of the people reporting SPD are males) raise questions regarding possible sex differences in SPD. Toward that end, some preliminary data based on 760 adults with SPD recruited online suggest there are no clinical sex differences in SPD (15), whereas other research in 245 university students suggests men with SPD may report more pleasure from the behavior and may pick at areas that are less noticeable to others (legs compared to face) (16). In a different study of university students (n=1916), men with SPD perceived themselves as significantly less attractive to others compared to women with SPD and reported fewer depressive symptoms than women with SPD (17). In one of the few neuroimaging studies to examine both men and women with SPD (21 women and 14 men), the researchers failed to identify any gender differences with regard to grey matter volumes in regions of interest in SPD (i.e. basal ganglia, orbitofrontal cortex, insula), but found that women with SPD reported more severe picking and more focused picking (18). If we examine sex differences in trichotillomania, arguably the disorder with the greatest phenomenological similarity to SPD, we find that men more commonly have later age of onset of behavior and less functional impairment due to their pulling than women (19–21).

The National Institute of Mental Health in the United States has stressed that the role of sex differences is important for an accurate interpretation of research findings, as sex may reflect different disease processes between women and men, and that these difference may facilitate the development of more precision interventions in both sexes. Thus, one approach to refining our treatment of SPD might be to better understand sex differences and their clinical associations. Here, our aim was to identify clinical and demographic measures associated with sex in a non-treatment-seeking sample of adults with SPD. Given the extant literature, we hypothesized that men with SPD would have a later age of onset of the disorder, fewer comorbidities, and less functional impairment than women with SPD.

## Methods

### Participants

Non-treatment seeking adults, ages 18-65 with a primary and current DSM-5 diagnosis of SPD (n=95; 17.9% male) were enrolled in a study regarding the clinical characteristics of SPD. Participants were recruited using flyers, online advertisements, and referrals. The only inclusion criterion was SPD as the person’s primary disorder. The only exclusion criterion was an inability to understand and consent to the study.

Data were collected at the University of Chicago from March 2017 to September 2018. The study, and consent processes, were approved by The University of Chicago Institutional Review Board. After participants were given a comprehensive explanation of study procedures and an opportunity to ask any questions, they provided written informed consent. This research was conducted in accordance with the principles of the Declaration of Helsinki. Participants were compensated 75 USD for time and travel, and were permitted to take rest breaks during the procedures if needed.

### Assessments

Participants were diagnosed using DSM-5 criteria. A semi-structured interview was used to acquire demographic information and data regarding the clinical characteristics of SPD. The interview included questions regarding age of onset of picking, intensity of urges, environmental or emotional triggers, and frequency and duration of the skin picking.

We used the Mini-International Neuropsychiatric Interview 7.0 (MINI 7.0) (22) to screen for co-occurring psychiatric disorders. Family medical and psychiatric history was also assessed in first-degree relatives of the participants (although family members were not interviewed).

The following clinical measures were used to assess symptom severity, anxiety, stress, and impulsivity.

*Clinical Global Impression – Severity scale (CGI-S)* (23). The CGI-Severity scale **is** a reliable and valid, 7-item scale used in this study to assess clinical severity of SPD symptoms. The CGI-Severity scale was scored from 1 = “not ill at all” to 7 = “among the most extremely ill”.

*Milwaukee Inventory for the Dimensions of Skin Picking (MIDAS)* (24). This 12-tem scale assesses automatic and focused picking styles.

*Sheehan Disability Scale (SDS)* (25). The SDS is a reliable, valid, 3-item, self-report scale that was used to assess how skin picking affected functioning in three areas of life: work/school, social/leisure activities, and home/family life. Scores on the SDS range from 0 to 30, with each question ranging from 0 (no disruption) to 10 (extreme disruption).

*Quality of Life Inventory* (QOLI) (26). The QOLI is a self-report scale consists of 16 domains of life: family, love, work, and children, health, self-esteem, goals and values, money, play, learning, creativity, helping, friends, relatives, home, neighborhood, and community. Individuals are asked to rate the importance of each domain along with how satisfied they are with that domain.

*Barratt Impulsiveness Scale 11 (BIS-11)* (27). The BIS-11 is a 30 question self-report questionnaire examining general impulsiveness as well as three second order domains: motor, non-planning, and attentional impulsiveness.

### Data analysis

Gender differences in the demographic and clinical characteristics, skin picking sites, and presence of comorbidities, were examined using analysis of variance for continuous variables and likelihood ratio chi-square tests for categorical variables. This being an exploratory study, and in view of the sample size, statistical significance was defined as p<0.05 uncorrected. All analyses were conducted using JMP Pro software.

## Results

### Participant characteristics

Ninety-five adults (mean age = 32.5 ± 11.9 years; 17.9% male) with primary DSM-5 SPD were included. The demographic and clinical variables are presented in Table 1. Participants had the following self-identified racial breakdown with no significant differences in demographic indicators between groups: 74.7% White/Caucasian, 5.3% Black/African-American, 4.2% Asian, 5.2% Biracial, and 6.3% Other. Men and women with SPD did not significantly on demographic measures (all p>0.10). Men and women with SPD also did not significantly differ on skin picking severity (moderate scores on the CGI), functional disability (mild-moderate disability), impulsivity (low levels) or quality of life (low quality of life). Men with SPD were significantly more likely to report a first-degree relative with skin picking or hair pulling.

Participants were asked about sites on their body where they picked. It could be a single site or multiple sites. These data are presented in Table 2. Men were significantly less likely to pick from their scalps, backs or breasts. The two groups did not differ in terms of other sites they picked. Overall, women picked from significantly more sites than men did.

Data from the MINI regarding comorbid conditions are presented in Table 3. Rates of current comorbid psychiatric conditions did not differ significantly between men and women with SPD.

## Discussion

This study examined potential sex differences in the clinical presentation of SPD in adults. Men and women with SPD had similar presentations in terms of severity of symptoms and impairment, and similar levels of impulsiveness. They also did not differ significantly in terms of occurrence of comorbidities, nor in terms of whether the picking tended to be more ‘automatic’ of ‘focused’. These findings indicate that SPD needs to be taken seriously irrespective of sex, due to high rates of impairment and comorbid conditions.

Having found men and women with SPD were similar on most measures, we did find evidence for some clinical differences in specific domains. Compared to women with SPD, men typically picked from fewer bodily sites. Additionally, men with SPD were less likely to pick at skin from their backs, scalp, and breast areas. The clinical significance of these results or the reason for them is unclear, since men and women had similar overall levels of symptom severity. This may suggest that while picking sites differ somewhat, overall the picking is similarly impairing, as also reflected in the quality of life and disability measures being similar across genders.

In terms of family history of other conditions, interestingly, men with SPD had higher rates of skin picking and/or trichotillomania in their first-degree relatives, but not notably higher rates of the other conditions examined (OCD and substance use disorders). There is virtually a complete absence of research into genes conferring vulnerability to SPD (28). A previous study examined heritability in female twin pairs, and estimated the heritability of SPD to be around 40% (29). Based on our finding of higher rates of SPD and trichotillomania in the first-degree relatives of men with SPD, we predict that heritability may be higher in men.

Several limitations should be noted in terms of this study. First, the sample was small, and so relatively subtle sex differences may not have been detected. Second, and in view of the sample size, findings should be considered tentative and in need of replication in larger studies. Third, we recruited non-treatment seeking participants and so it is not yet known whether these findings would generalize to clinical settings. Nonetheless, we feel it is important to draw attention to potential similarity and differences in the presentation of SPD as a function of sex.

In conclusion, both men and women with SPD showed similar functional impairment, rates of mainstream mental disorders, and impulsiveness. However, differences in picking sites as well as in family history of picking and trichotillomania were identified between sexes, highlighting the need for more research to be conducted, including in the areas of heritability and genetics.

## Figures and Tables

**Table 1:** Comparison of Men and Women with SPD Based on Demographic and Clinical Measures.

	Men (N=17)	Women (N=78)	F	p
Age, years	32.6 (13.0)	32.5 (11.7)	0.001	0.97
Racial-ethnic group, non-White Caucasian #	3 [17.7%]	21 [26.9%]	7.533c	0.11
Education level	5.9 (1.3)	5.9 (1.3)	<0.001	0.986
Age at onset of SPD, years	15.6 (8.8)	13.2 (7.9)	1.2469	0.267
MIDAS Focused	16.1 (4.9)	18.1 (5.0)	1.897	0.172
MIDAS Automatic	19.6 (4.7)	18.2 (4.3)	1.2926	0.2586
Clinical Global Impression-Severity	4.0 (0.5)	4.3 (0.8)	2.428	0.1226
Sheehan Disability Scale	6.4 (5.8)	8.5 (7.4)	1.202	0.276
BIS Attentional	16.3 (4.2)	16.2 (4.0)	<0.001	0.986
BIS Motor	21.6 (4.3)	21.2 (3.8)	0.1396	0.7096
BIS Non-planning	24.7 (5.2)	23.1 (5.0)	1.2579	0.2651
QOL T-score	41.8 (14.5)	44.8 (12.8)	0.7011	0.4046
Participants with at least one first-degree relative(s) with SPD or trichotillomania, N [%]	3 [17.7%]	1 [1.3%]	6.625	0.0101
Participants with at least one first-degree relative(s) with Obsessive-Compulsive Disorder, N [%]	1 [5.9%]	1 [1.3%]	1.094	0.2957
Participants with at least one first-degree relative(s) with a substance use disorder, N [%]	2 [11.8%]	14 [17.94%]	2.643	0.450

# For simplicity, data are presented in binary form, but likelihood ratio test examined breakdown across all racial-ethnic subgroups. c: Likelihood ratio chi-square test. MIDAS: Milwaukee Inventory for Dimensions of Skin Picking; BIS: Barratt Impulsivity Scale; QOL: Quality of Life Inventory.

**Table 2:** Comparison of Men and Women with SPD Based on Skin Picking Sites.

	Men (N=17)	Women (N=78)	LR	p
Picked only at a single site, N [%]	8 [47.1%]	13 [16.7%]	6.569	0.0104
Picked at 1-2 sites, N [%]	13 [76.5%]	24 [30.8%]	12.177	<0.001
Picked at 3 or more sites, N [%]	3 [17.6%]	49 [62.8%]	12.055	0.001
Scalp, N [%]	2 [11.8%]	32 [41.0%]	5.998	0.0143
Face, N [%]	8 [47.1%]	54 [69.2%]	2.905	0.0883
Back, N [%]	1 [5.9%]	29 [37.2%]	7.943	0.0048
Arms, N [%]	6 [35.3%]	36 [46.2%]	0.678	0.4103
Legs, N [%]	3 [17.7%]	28 [35.9%]	2.308	0.1287
Breast area, N [%]	0 [0.0%]	15 [19.2%]	6.501	0.0108
Pubic area, N [%]	0 [0.0%]	6 [7.7%]	2.453	0.1173
Arm pits, N [%]	0 [0.0%]	7 [9.0%]	2.879	0.0897
Other, N [%]	8 [47.1%]	37 [47.4%]	0.001	0.9775

LR: Likelihood ratio chi-square test. Note: Examination of descriptions for ‘other’ indicated this category commonly comprised fingers including both finger skin itself and/or cuticles. Note: in some cases proportions may not add up to 100% due to blank responses to some questions.

**Table 3:** Comparison of Men and Women with SPD Based on Current Comorbidities.

	Males (N=17)	Females (N=78)	LR	p
Had only one comorbid disorder, N [%]	1 [11.1%]	15 [20.6%]	0.577	0.448
Had multiple comorbid disorders, N [%]	4 [23.5%]	27 [37.0%]	0.111	0.739
Obsessive-Compulsive Disorder, OCD, N [%]	2 [11.8%]	5 [6.4%]	0.522	0.4699
Body dysmorphic disorder, BDD, N [%]	0 [0.0%]	4 [5.3%]	1.477	0.2243
Generalized anxiety disorder, GAD, N [%]	5 [29.4%]	27 [35.1%]	0.423	0.5155
Social phobia, N [%]	0 [0.0%]	4 [5.3%]	1.203	0.2728
Specific phobia, N [%]	0 [0.0%]	4 [5.2%]	1.188	0.2758
Panic disorder, N [%]	2 [11.8%]	4 [5.3%]	0.838	0.36
Agoraphobia, N [%]	0 [0.0%]	0 [0.0%]	0	>.999
Depression, N [%]	4 [40.0%]	32 [41.0%]	0.004	0.9504
Bipolar Disorder, N [%]	1 [5.9%]	1 [1.4%]	1.026	0.3112
Tourette’s Disorder, N [%]	0 [0.0%]	2 [2.6%]	0.494	0.4819
ADHD, N [%]	2 [16.7%]	5 [6.7%]	1.148	0.284
PTSD, N [%]	0 [0.0%]	5 [6.6%]	2.081	0.149
Anorexia nervosa, N [%]	0 [0.0%]	3 [4.0%]	1.167	0.2799
Bulimia nervosa, N [%]	0 [0.0%]	1 [1.3%]	0.384	0.5352
Binge-eating disorder, N [%]	0 [0.0%]	3 [4.0%]	1.167	0.2799
Alcohol use disorder, N [%]	0 [0.0%]	1 [1.3%]	0.406	0.5239
Substance use disorder, N [%]	0 [0.0%]	2 [2.6%]	0.817	0.336

LR: Likelihood Ratio chi-square test; ADHD: Attention-deficit hyperactivity disorder; PTSD: Post-traumatic stress disorder.
